# Oligomerization of Silicic Acids in Neutral Aqueous Solution: A First-Principles Investigation

**DOI:** 10.3390/ijms20123037

**Published:** 2019-06-21

**Authors:** Xin Liu, Cai Liu, Changgong Meng

**Affiliations:** State Key Laboratory of Fine Chemicals, School of Chemical Engineering, Dalian University of Technology, No. 2, Linggong Road, Dalian 116024, China; kikikiss@aliyun.com (C.L.); cgmeng@dlut.edu.cn (C.M.)

**Keywords:** zeolite, ortho-silicic acid, oligomerization, first-principles, nucleation

## Abstract

Crystallite aluminosilicates are inorganic microporous materials with well-defined pore-size and pore-structures, and have important industrial applications, including gas adsorption and separation, catalysis, etc. Crystallite aluminosilicates are commonly synthesized via hydrothermal processes, where the oligomerization of silicic acids is crucial. The mechanisms for the oligomerization of poly-silicic acids in neutral aqueous solution were systematically investigated by extensive first-principles-based calculations. We showed that oligomerization of poly-silicic acid molecules proceeds through the lateral attacking and simultaneously proton transfer from the approaching molecule for the formation of a 5-coordinated Si species as the transition state, resulting in the ejection of a water molecule from the formed poly-silicic acid. The barriers for this mechanism are in general more plausible than the conventional direct attacking of poly-silicic acid with reaction barriers in the range of 150–160 kJ/mol. The formation of linear or branched poly-silicic acids by intermolecular oligomerization is only slightly more plausible than the formation of cyclic poly-silicic acids via intramolecular oligomerization according to the reaction barriers (124.2–133.0 vs. 130.6–144.9 kJ/mol). The potential contributions of oligomer structures, such as the length of the linear oligomers, ring distortions and neighboring linear branches, etc., to the oligomerization were also investigated but found negligible. According to the small differences among the reaction barriers, we proposed that kinetic selectivity of the poly-silicic acids condensation would be weak in neutral aqueous solution and the formation of zeolite-like structures would be thermodynamics driven.

## 1. Introduction

Zeolites are crystalline silicates with well-defined microporous structures and superior thermo- and chemo-stability. They are widely used in many industrial fields of great significance, including as porous support or catalysts in conversion of chemicals [1,2,3,4,5], as adsorbents for removal and recovery of heavy metal ions from water [6,7,8] and for selective gas adsorption and separation [9], etc [10,11,12,13]. Zeolites for industrial applications are conventionally synthesized through hydrothermal processes, where the oligomerization of poly-silicic acids is crucial [3]. People have come to realize the significant roles of the concentration of silicate precursors, pH values of the reaction mixture, and the reaction temperatures to the formed framework structure and crystallinity of the zeolite materials [14,15,16]. However, due to the complexity of the hydrothermal reaction and the lack of suitable experimental techniques, limited knowledge has been gained at molecular level on the oligomerization of poly-silicic acids, especially at the initial nucleation and during the growth of zeolite materials, even though they are crucial [17,18,19,20]. These make it still challenging to develop synthesis routes for controlled fabrication of zeolite materials with desired structures for industrial applications [4,15,19,21,22,23,24]. 

Considerable theoretical efforts have been put to understand the oligomerization of silicic acids [22,25]. The reaction mixtures for zeolite synthesis are conventionally prepared in acidic or basic condition. Catlow et al. investigated the condensation of protonated silicic acid molecules in water/alkoxide and methanol/alkoxide [26,27,28,29]. They showed that the condensation may take place through an S_N_2-like mechanism by directly attaching of hydroxyl of the non-protonated silicic acid to the pronated one in the reaction reverse to the protonated hydroxyl to form a 5-coordinated Si species followed by detaching of a water molecule. They also showed that the lateral attack of the hydroxyl of the non-protonated silicic acid to the pronated one nearly vertical to the direction of the detaching water is also possible but with an activation energy of 17.3 kcal/mol. Trinh et al. proposed that the reaction in basic solution initiates with deprotonated silicic acid through a 5-coordinated Si species would be more plausible, and the reaction barriers for formation and dissociation of the 5-coordinated Si species are 57 and 66 kJ/mol, respectively [30]. They also proposed that the condensation in neutral solution would take place through a one-step lateral attaching mechanism crossing a transition state where the formation of the 5-coordinated Si species and proton transfer to form water take place simultaneously at a barrier of 127 kJ/mol. [30]. This mechanism was also supported by the work of Henschel et al. and Hu et al. [31,32]. Thomson et al. investigated the mechanism for condensation of silicic acid to form linear dimer and trimer, cyclic trimer and tetramer, prismatic hexamer and cubic octamer [33]. They concluded that under neutral conditions, the condensation should proceed through a single-step, S_N_2-like mechanism where the formation of the 5-coordinated Si species and proton transfer for water formation take place simultaneously, though the proton catalyzed processes would take place with a two-step mechanism through the 5-coordinated Si species as an intermediate or a single-step mechanism as that for the neutral solution. Researchers also proposed that the oligomerization of silicic acid can be affected by several factors, such as pH, the dielectric constant of the reaction mixture, etc. Tossell investigated the free energy change for the oligomerization of silicic acid with advanced quantum mechanics techniques and found that condensation in water is weakly endothermic and the reaction may prefer to take place at elevated temperature and pressure in a solution with low dielectric constant [34]. Mora-Fonz et al. calculated the free energy of all potential linear and ring polysilicic acids and their deprotonated forms containing less than five Si atoms and proposed that the thermodynamic of oligomerization correlates strongly with the pH. They concluded that the formation of ring structures is plausible as compared with the linear structures and is pH driven [35,36]. White et al. investigated the thermodynamics for the deprotonation and condensation of silicic acids and they proposed that there would be a strong correlation between the reaction exothermicity and the pH value of the reaction mixture [37]. People also investigated the mechanism for the hydrolysis of silica that is the reverse to the oligomerization. Xiao et al. investigated the gas phase hydrolysis of silicic acid dimer in existence of OH^-^ and they showed that water adsorbs firstly at one of the Si atoms for the formation of a 5-coordinated Si species with a barrier of 79 kJ/mol, which dissociates into two silicic acid molecules by crossing a barrier of 19 kJ/mol [38]. Pelmenschikov [39] and Criscenti [40] investigated the hydrolysis of silica in neutral and acidic solutions, and both of them found that the barrier for the formation of Si–O–Si is about 120 kJ/mol. These results provide theoretical evidence on the oligomerization of poly-silicic acid and also stimulate several attempts to investigate the growth of poly-silicic acids in reaction condition with Monte Carlo simulations [41,42,43,44]. 

It is generally believed that oligomerization of silicic acid during the hydrothermal process is crucial for nucleation and growth of the zeolite framework [45]. Due to the complexity of the structures of potential oligomers of silicic acid, only small oligomers containing less than six Si atoms has been investigated, and the results are still hard to render the zeolite formation and growth mechanism [25,26,27,28,29,30,31,32,33,34,35,36,37,38,39,40,41,42,43,44,45]. The oligomerization of poly-silicic acids was considered selective and the formation of some poly-silicic acids as precursors for zeolite framework would be either kinetics- or thermodynamics-driven [18,19,20]. However, these aspects of the reaction mechanism have not been visited. Inspired by these pioneer works, we performed extensive first-principles-based calculations to highlight the mechanisms, thermodynamics, and kinetics for the oligomerization of poly-silicic acids in neutral aqueous solution.

## 2. Results and Discussions

**Figure 1:**
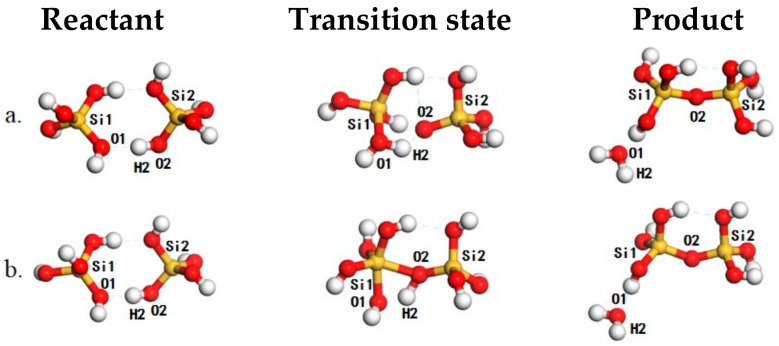
The structures of reaction species for the dimerization of ortho-silicic acid, including reactant (**left panel**), transition state (**middle panel**) and product (**right panel**) for the anionic-attack mechanism (AAM) (**a**) and the “molecular attack” mechanism (MAM) (**b**) mechanisms. The Si, O and H atoms are in yellow, red, and white, respectively.

In general, the nucleation and growth of zeolites and colloidal silica can be considered as oligomerization reactions among poly-silicic acid molecules. The processes can be described by networks of condensation reactions among monomers and oligomers of ortho-silicic acid. We firstly investigated the dimerization of ortho-silicic acid (Figure 1). 

When two ortho-silicic acid molecules meet, a hydrogen-bonded complex (Figure 1, left panel) is formed. The formation of this hydrogen-bonded complex is exothermic by −16 kJ/mol with respect to freestanding ortho-silicic acid molecules and is comparable with the interaction with H_2_O (−19 kJ/mol). In this structure, the O1–H2 distance is 1.81 Å and is typical for a strong hydrogen bond. The O2–H2 distance is 0.99 Å corresponding to a hydrogen-bond distorted Si–OH. Then, the reaction may proceed with a proton transfer from O2 to O1 accompanying the attacking of O2 to Si1 leading to the formation of the transition state (Figure 1a, middle panel). During this process, the O2–H2 distance is elongated to 1.53 Å while the O1–H2 distance is shortened to 1.05 Å, suggesting the formation of the O1–H2 bond and the dissociation of O2–H2 bond. Simultaneously to these, the Si1–O2 distance is also shortened from 3.97 to 2.20 Å, showing the potential for the formation of the Si–O bond. As driven by the electrostatic interaction between the Si1 and O2, the hydrogen-bonded complex would cross a reaction barrier of 133 kJ/mol (Figure 1a, middle panel) to reach the product with the ejection of the formed H_2_O from the first coordination shell of Si1 (Figure 1, right panel) and the calculated imaginary frequency is -294.4 cm^−1^. As the proton transfer is prior to the formation of the Si–O bond, we named it anionic-attack mechanism (AAM). The calculated reaction barrier compares well with that reported by Trinh et al. [30,41]. Alternatively, the reaction may also proceed without the proton transfer by directly attacking Si1 with the hydroxyl connected to Si2 in the reactant. At the corresponding transition state (Figure 1b, middle panel) that corresponds to a 5-coordinated Si species, the Si1–O2 distance is shorted to 1.97 Å and is even 0.23 Å shorter than that in the anionic mechanism. Furthermore, the O2–H2 distance is also elongated to 1.16 Å while O1–H2 distance is changed to 1.29 Å. In this sense, the structure change to the O2–H2 and O1–H2 is not that significant as in the anionic mechanism, and bonding character of O2–H2 is largely preserved. As the hydroxyl in ortho-silicic acid is less charged without proton transfer, the O2–Si1 electrostatic interaction is much weaker as compared with that in the AAM, and reaction barrier is ~159 kJ/mol. The corresponding imaginary frequency for this transition state is −997.8 cm^−1^. After the transition state, proton transfer of H2 from O2 to O1 may take place, and the formed H_2_O is detached and bonds the dimer with hydrogen bond (Figure 1, right panel). As the proton transfer takes place later than the formation of the Si–O bond, we named it as “molecular attack” mechanism (MAM).According to the direction of the detached H_2_O that is vertical to the formed Si-O bond, both AAM and MAM can be classified as variants to the previously proposed lateral attacking mechanism [26,27,28,29,30]. These findings highlight the important role of proton transfer in the oligomerization of poly-silicic acids. In AAM, the proton transfer leads to the exposure of the negatively charged O2 to the positively charged Si1 and enhances the electrostatic interaction between them as the driving force for the formation of the Si–O bond while also balances the instability induced by charge separation. This may also ease the dissociation of the 5-coordinated Si species for the ejection of the formed H_2_O. In MAM, the O2 is less charged and the reaction lacks a driving force while the remnant proton at O2 may also destabilize the 5-coordinated Si-species. These account for the relatively high reaction barrier of 159 kJ/mol along MAM compared with that for the AAM. 

We then moved on to investigate the potential mechanisms for oligomerization among poly-silicic acids. When two oligomers of ortho-silicic acid meet, the reaction may take place between two silicic acid groups. This makes it reasonable to simplify one of the oligomers to an ortho-silicic acid molecule. As for the reactions following the AAM mechanism, proton transfer may take place within the hydrogen-bonded complexes. The calculated deprotonation free energy change of ortho-silicic acid and its dimer are −63 and −95 kJ/mol, respectively, in excellent agreement with those reported by Trinh [30] and Mora-Fonz [35], showing that the plausible proton transfer may take place from the poly-silicic acid to the ortho-silicic acid. 

We classified the structure of the oligomers of ortho-silicic acid as linear ($T_{n}^{m}$) and cyclic oligomers ($C_{n}^{m}$). The degree of oligomerization along the major framework of the oligomer was described by subscript n while the branched structures were described by the superscript m. For example, $C_{4}^{2}$ describes a four-ring oligomer with two neighboring branches. The superscript is omitted when m is zero. The energy profiles for the formation of linear oligomers were collected in Table 1. 

**Figure 2:**
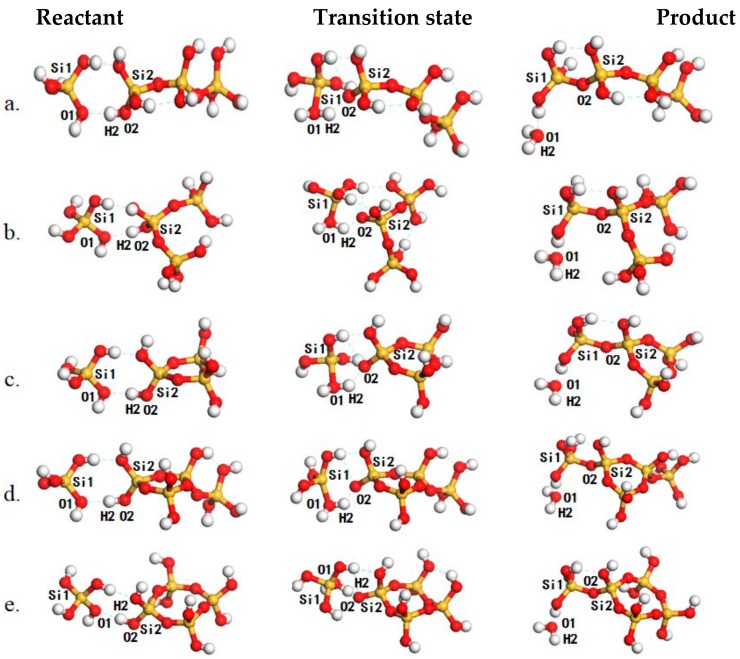
The structures of reaction species, including reactants (**left panel**), transition states(middle panel) and products (**right panel**) for oligomerization for the formation of $T_{4}$ (**a**), $T_{3}^{1}$ (**b**), $C_{3}^{1}$ (**c**), $C_{3}^{2}$ (**d**) and $C_{4}^{1}\;$ (**e**). The Si, O and H atoms are in yellow, red and white, respectively.

The oligomerization for the formation of $T_{4},T_{3}^{1}$, $C_{3}^{1}$, $C_{3}^{2},$ and $C_{4}^{1}$ through AAM mechanism were selected from Table 1 to discuss the potential impact of the structure of the oligomers on the reaction (Figure 2, Table 1). The oligomerization of $T_{1}$ and $T_{3}$ leads to the formation of $T_{4}$ (Entry 3, Table 1 and Figure 2a). A hydrogen-bonded complex is formed between $T_{1}$ and $T_{3}$ and this complex is stabilized by two hydrogen bonds both at 1.81 Å. Then, the reaction proceeds with the approaching of O2 to Si1. In the transition state, the Si1–O2 distance is 2.21 Å while that of Si1–O1 is elongated to 1.84 Å, showing the tendency to detaching the H_2_O. The reaction barrier is 130.2 kJ/mol. However, the oligomerization of $T_{1}$ and $T_{3}$ may also lead to the formation of $T_{3}^{1}$ (Entry 4, Table 1 and Figure 2b). In the corresponding transition state, the H2–O1 distance is decreased to 1.05 Å while the H2–O2 distance is increased to 1.51 Å. The reaction proceeds by crossing a reaction barrier of 124.2 kJ/mol. As $T_{3}$ can condense by intra-molecular oligomerization to form $C_{3}$. The major difference between the formation of $T_{3}^{1}$ and $C_{3\;}^{1}$(Entry 8, Table 1 and Figure 2c) is that the Si group in $C_{3}$ is confined by the adjacent Si–O bonds along the cyclic structure. Resulting from this confinement, the barrier for the formation of $C_{3}^{1}$ is 127.9 kJ/mol, slightly higher than that for the formation of $T_{3}^{1}$. The impact of the existence of an adjacent monomer branch to the oligomerization was also investigated (Entry 12, Table 1, and Figure 2d), but the barrier for $C_{3}^{2}$ was found to be higher by only 1.6 kJ/mol compared with the formation of $C_{3}^{1}$. The formation of $C_{3}^{1}$ and $C_{4}^{1}$ were also investigated (Entry 9, Table 1, and Figure 2e) to highlight the impact of the Si–O–Si–O–Si angle to the condensation, the barrier for $C_{4}^{1}$ is found only 0.6 kJ/mol higher than that for $C_{3}^{1}$.

We then fell back to Table 1 to generalize the findings above. For the formation of linear oligomers, ${\;T}_{2}$, $T_{3}$, $T_{4}$, $T_{5}$, $T_{6},$ and $T_{7}$ (Entries 1–3 and 5–7, Table 1) through the AAM mechanism, the energy barriers are 133.0, 131.9, 130.2, 131.6, 132.6, 131.8 kJ/mol, respectively. The barrier for the formation of the branched linear oligomers, $T_{3}^{1}$ (Entry 4, Table 1), is only 124.2 kJ/mol, ~6.0 kJ/mol lower than that for the linear oligomers with the same rate of oligomerization. The formation of branched cyclic oligomers, $C_{3}^{1}$, $C_{4}^{1},\;C_{5}^{1},$ and $C_{6}^{1}$ (Entries 8–11, Table 1) is in the range from 126.5 to 132.0 kJ/mol, slightly lower than the formation of linear oligomers, showing the competition with the formation of linear oligomers. The slight difference in the reaction barriers for the formation of the $C_{3}^{1}$, $C_{4}^{1},\;C_{5}^{1}$ and $C_{6}^{1}$ can be attributed to the difference in the –O–Si–O– angle in the cyclic oligomers. The existence of an adjacent monomer branch to the oligomerization was also investigated and the formation barriers for $C_{3}^{2}$, $C_{4}^{2},\;C_{5}^{2}$ and $C_{6}^{2}$ (Entries 12–15) falls in the range from 126.0 to 129.5 kJ/mol, showing the less significant role of the adjacent branch to the further oligomerization. According to the small variation (within 10 kJ/mol) of reaction barriers with respect to the oligomer structure, these oligomers would exhibit similar reactivity to further oligomerization, and the kinetic selectivity of oligomerization of poly-silicic acids would be weak.

**Figure 3:**
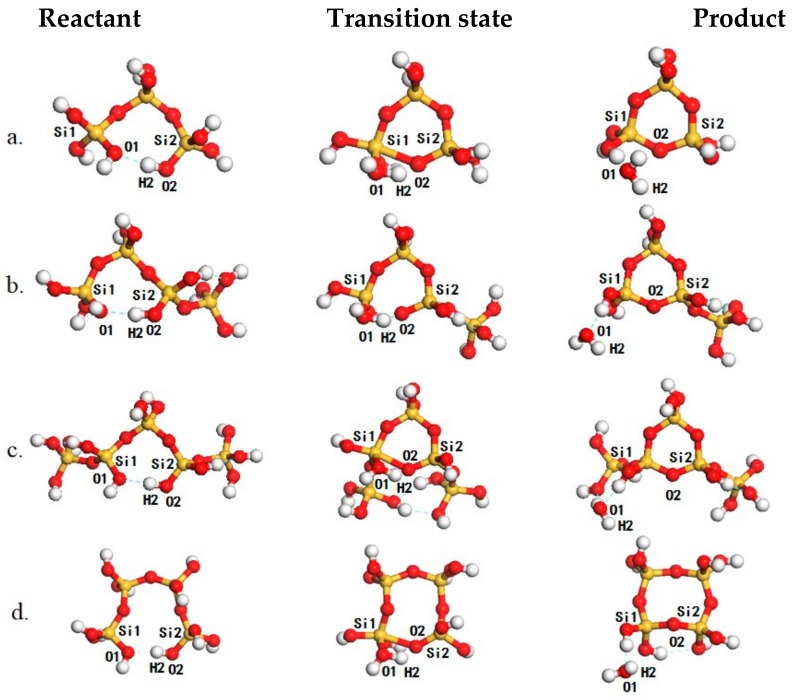
The structures of reaction species, including reactants (left panel), transition states (**middle panel**) and products (**right panel**) for oligomerization for the formation of $C_{3}$ (**a**), $C_{3}^{1}$ (**b**), $C_{3}^{2}$ (**c**) and $C_{4}^{1}$ (**d**). The Si, O and H atoms are in yellow, red and white, respectively.

We then investigated the intramolecular oligomerization of poly-silicic acids for the formation of cyclic poly-silicic acid oligomers (Table 2 and Figure 3). As there are plenty of hydroxyl groups in the oligomers, long linear oligomers may exhibit a distorted structure with intramolecular hydrogen bonds which can balance the distortion and provide additional stability. For example, the most plausible structure of $T_{3}$ (Figure 3a, left panel) is stabilized by an intramolecular hydrogen bond formed one hydroxyl group at each end. While O2–H2 is approaching Si for the formation of the Si–O2 bonds, the O2–H2 distance is increasing and the O1–H2 distance is decreasing. In the transition state (Figure 3a, middle panel), the H2–O2 distance is increased to 1.47 Å while the O1–H2 is decreased to 1.07 Å and is typical for strong hydrogen bonds. Due to the distortion within the cyclic structure, the instability of the 5-coordinated Si species and the charge separation induced by proton transfer within the hydrogen-bonded network, the reaction barrier is 131.9 kJ/mol (Entry 1, Table 2). Similarly, $T_{4}$ may adapt a conformation like that of $T_{3}$ in its most plausible form (Figure 3b, left panel) for the formation of a branched $C_{3}$ ($C_{3}^{1}$, Figure 3b, right panel) or a cyclic conformation with a head-to-tail hydrogen bond (Figure 3d, left panel) for the intramolecular oligomerization to form a $C_{4}$ (Figure 3d, right panel). The calculated reaction barriers for these two processes are 130.6 (Entry 5, Table 2) and 141.8 (Entry 2, Table 2) kJ/mol, respectively. In the same way, $T_{5}$ may also adapt a conformation like $T_{3}$ by forming a hydrogen bond between two hydroxyl attached to Si atoms next to the two ends (Figure 3c, left panel) to form a cyclic oligomer with two branch monomers ($C_{3}^{2}$, Figure 3c, right panel) with a barrier of 141.8 kJ/mol (Entry 9, Table 2). Alternatively, $T_{5}$ can also transform to the head-to-tail conformation to form $C_{5}$ (Entry 3, Table 2), or a conformation similar to the head-to-tail conformation to form $C_{4}^{1}$ (Entry 6, Table 2). The calculated barriers for these two oligomerizations are 140.3 and 140.4 kJ/mol, respectively. The differences in the reaction barriers starting from different conformers of $T_{5}$ can be attributed to the torsion within the initial conformer, the hydrogen-bonded network for proton transfer and proton transfer induced charge separation. Except for $C_{3}^{1}$ and $C_{3}$, the calculated barriers for the formation of cyclic oligomers fall in the range from 140 to 145 kJ/mol showing that their formations are not kinetically selective. Due to the large torsion within $C_{3}$, the calculated barriers for $C_{3}^{1}$ and $C_{3}$ are 131.9 and 130.6 kJ/mol and are comparable to the formation barriers of those linear oligomers showing that the $C_{3}$-based oligomer may act as active intermediates for further oligomerization of poly-silicic acids. This is supported by the experimental fact that there are no $C_{3}$-based building blocks in the reported zeolite structure database. 

We also calculated the energy profile for the formation of linear and cyclic oligomers through the MAM mechanism (Table 3 and Table 4). It should be noted that, due to the small structure variation to adapt the MAM mechanism, the calculated exothermicity and free energy change for specific reactions may vary from those for the AAM mechanism. The calculated reaction barriers for linear oligomers fall in the range from 150 to 160 kJ/mol (Table 3) while those for cyclic oligomers fall in the range from 130 to 150 kJ/mol (Table 4), and vary within 20 kJ/mol. These reaction barriers are ~30 kJ/mol higher than those following the AAM mechanism (Table 1 and Table 2), showing the dominant role of proton transfer in the oligomerization of poly-silicic acids. 

These oligomerization reactions are slightly exothermic according to calculated ΔE, ΔH_298K_, ΔH_450K,_ ΔG_298K_ and ΔG_450K_ (Table 1, Table 2, Table 3 and Table 4) and the maximum exothermicity are 20.5, 20.9, 19.2, 31.3 and 42.0 kJ/mol, in terms of reaction energy, enthalpy and free energy change at 298 and 450 K, respectively. The only available experimental thermodynamics data concerning silicic acid oligomerization is the experimental equilibrium constant that is ~20 [46]. This value corresponds to free energy of −7 kJ mol^−1^ and compares well with our result of −8.5 kJ/mol (Table 1) and those reported by Mora-Fonz [35,36,47] and Schaffer [33]. According to the difference in ΔH _298K_, ΔH _450K,_ ΔG_298K_ and ΔG_450K_, these oligomerization reactions would be more plausible at elevated temperatures. The small enthalpy and free energy change for the oligomerizations and the similar reaction barriers indicate that these reactions would reach an equilibrium state, and the formation of crystalline silicate structures would be thermodynamics driven. This is in agreement with experimental findings and may help clarify that zeolite nucleation and growth is not directly from specific oligomers. Previously, Putz et al. developed the concept of chemical hardness to describe chemical reactivity. We believe this would be a future direction to understand the oligomerization mechanism of poly-silicic acids and we are working on these [48,49,50,51]. 

The reaction conditions may have an impact on the oligomerization. However, the gap between first-principles-based calculations and real experiments is that the impact of reaction conditions, such as pH and concentration of reactants, cannot be treated accurately. Previous discussions on the impact of pH on the existence form and variation of poly-silicic acids were based on the direct reaction of silicic acid anions. Obviously, this cannot lead to quantitative results. The limited success of first-principles-based calculations with temperature is due to the fact that the thermodynamics information about the reaction system can be derived from partition functions. The proposed AAM and MAM mechanisms highlight the significant role of proton transfer among the reacting poly-silicic acid molecules to the oligomerization. As the proton concentration would be sensitive to the pH of the reaction mixture, increasing the pH may help to promote the proton transfer or directly turn the poly-silicic acid molecules into the corresponding anions. In this way, the reaction takes place between anions and poly-silicic acid molecules and the mechanism also changes into a two-step mechanism through the formation of a 5-coordinate Si species as the intermediate. It should be noted that with anion as the approaching group, the reaction barrier would be ~60 kJ/mol and is much lower than those for the proposed mechanisms. Further, the solubility of orth-silicic acid is very low in a neutral aqueous solution at room temperature and so as that for other poly-silicic acids. The increase of pH may help the solvation and ionization of these precursors and raises the concentration of poly-silicic anions in the aqueous phase. These processes should be in equilibrium with the oligomerization and further the evolution of the oligomers. Previous experimental reports suggest that the variation of pH may shift the oligomer distribution and is in reasonable agreement with our proposal. Rising reaction temperature may help to increase the concentration of silicic acid in the aqueous solution phase. According to calculated free energy change at 298 and 450 K, the oligomerizations for the formation of cyclic and linear oligomers are all exothermic and the exothermicity increases with the temperature. In this sense, rising the reaction temperature may also accelerate the oligomerization to equilibrium.

## 3. Theoretical Methods

First-principles-based calculations were carried out to investigate the oligomerization mechanism of poly-silicic acid containing less than nine Si atoms. The calculations were performed with the generalized gradient approximation (GGA) functional developed by Becke, Lee, Yang and Parr (BLYP) [52,53] and full-electron DNP basis set in the DMol^3^ code [54,55]. The orbital cutoff was set as 4.6 Å for all types of atoms, and the potential interactions with solvent were handled with COSMO approach [56,57,58]. Geometry optimization convergence thresholds were set as 1 × 10^−6^ Hartree, 1 × 10^−3^ Hartree/Å and 1 × 10^−3^ Å for energy, force, and displacement, respectively. The convergence criterion for energy calculations was set as 1 × 10^−7^ Hartree. The transition states were located with complete LST/QST method according to the optimized structures of reactants and products and were confirmed by frequency calculations [59]. The energy barrier (ΔE_act_) of each reaction was calculated as the difference in electronic energy between the reactant and transition state. The free energy change (ΔG) of each reaction was calculated as the difference in free energy between the reactant and the product and includes the contribution from electronic, rotational, vibrational and transitional partition functions, and solvation, using standard statistical mechanics methods at 298 K and 450 K [60]. We estimated the Si–O and O–H distances and small water cluster. The Si–O distances are in the range of 1.64–1.68 Å with an average bond distance of 1.66 Å, while the O–H distances are in the range of 1.00 to 0.98 Å, with the average at 0.98 Å. The calculated O–H distances within water cluster are in the range from 1.00–0.98 Å with the average at 0.98 Å. The O–H distance is 0.98 in a water molecule. The interactions among water and reaction species broadened the distribution of Si–O and O–H distances and the distribution of O–H distance of H_2_O and silicic acid may overlap.

## 4. Conclusions

The oligomerization of silicic acids is crucial for the nucleation and growth of zeolites from the reaction mixture during the hydrothermal process. By using a neutral aqueous solution as a model system, the mechanisms for the condensation of silicic acids were systematically investigated by extensive first-principles-based calculations. We showed that silicic acid oligomerization proceeds through the lateral attacking and simultaneous proton transfer from the approaching silicic acid for the formation of a 5-coordinated Si species resulting in the formation of a water molecule that detaches from the poly-silicic acid. The barriers for this mechanism are in general more plausible than the conventional direct attacking of silicic acid with reaction barriers in the range of 150–160 kJ/mol. The formation of linear or branched poly-silicic acids by intermolecular oligomerization is only slightly more plausible than the formation of cyclic poly-silicic acids via intramolecular condensation according to the reaction barriers (124.2–133.0 vs. 130.6–144.9 kJ/mol). The potential contributions of oligomer structures, such as the length of the linear oligomers, ring distortions, and neighboring linear branches, etc., to the oligomerization were also investigated but found negligible. The small enthalpy and free energy change and the small differences among the reaction barriers for the oligomerizations indicate that these reactions would be in equilibrium, the kinetic selectivity of the poly-silicic acids oligomerization would be weak in neutral aqueous solution and the formation of crystalline silicate structures would be thermodynamics-driven.

## Figures and Tables

**Table 1:** Energy profile (kJ/mol) for the formation of linear oligomers through the AAM mechanism.

Entry	Reactions	ΔE_act_ *^a^*	ΔE *^b^*	ΔG_298K_ *^c^*	ΔG_450K_ *^c^*	ΔH_298K_ *^d^*	ΔH_450K_ *^d^*	If *^e^*
1	$T_{1}$ + $T_{1}$ → $T_{2}$ + $H_{2}O$	133.0	−3.0	−8.5	−11.4	−3.0	−2.3	−294.4
2	$T_{1}$ + $T_{2}$ → $T_{3}$ + $H_{2}O$	131.9	−6.3	−31.3	−42.0	−11.1	−9.5	−262.3
3	$T_{1}$ + $T_{3}$ → $T_{4}$ + $H_{2}O$	130.2	−4.9	−14.4	−18.8	−6.0	−5.1	−254.3
4	$T_{1}$ + $T_{3}$ → $T_{3}^{1}$ + $H_{2}O$	124.2	−2.4	−6.2	−9.2	−0.6	−0.3	−252.8
5	$T_{1}$ + $T_{4}$ → $T_{5}$ + $H_{2}O$	131.6	−3.0	−25.6	−35.8	−6.1	−4.9	−265.5
6	$T_{1}$ + $T_{5}$ → $T_{6}$ + $H_{2}O$	132.6	−2.0	−24.9	−35.1	−5.5	−4.0	−279.2
7	$T_{1}$ + $T_{6}$ → $T_{7}$ + $H_{2}O$	131.8	0.8	−8.1	−12.3	−0.2	0.5	−282.7
8	$T_{1}$ + $C_{3}$ → $C_{3}^{1}$ + $H_{2}O$	127.9	−1.8	−8.7	−12.5	−1.8	−0.9	−235.8
9	$T_{1}$ + $C_{4}$ → $C_{4}^{1}$ + $H_{2}O$	128.5	−2.6	−28.5	−39.2	−8.4	−6.5	−250.7
10	$T_{1}$ + $C_{5}$ → $C_{5}^{1}$ + $H_{2}O$	132.0	−4.6	−16.4	−21.4	−7.2	−6.0	−253.2
11	$T_{1}$ + $C_{6}$ → $C_{6}^{1}$ + $H_{2}O$	126.5	0.0	−16.9	−24.9	−1.8	−0.3	−252.8
12	$T_{1}$ + $C_{3}^{1}$ → $C_{3}^{2}$ + $H_{2}O$	129.5	−3.6	−1.8	−4.0	2.5	2.1	−227.8
13	$T_{1}$ + $C_{4}^{1}$ → $C_{4}^{2}$ + $H_{2}O$	129.3	−1.6	−10.1	−14.7	−1.4	−0.8	−220.2
14	$T_{1}$ + $C_{5}^{1}$ → $C_{5}^{2}$ + $H_{2}O$	126.6	−11.4	−8.4	−7.7	−9.5	−9.8	−273.4
15	$T_{1}$ + $C_{6}^{1}$ → $C_{6}^{2}$ + $H_{2}O$	126.0	2.7	−7.2	−12.5	2.6	3.5	−257.0

*^a^* The energy barrier for the oligomerization. *^b^* The exothermicity for the oligomerization. *^c^* The free energy change of the oligomerization at 298 K and 450 K. *^d^* The entropy change of the oligomerization at 298 K and 450 K. *^e^* Imaginary frequencies obtained from first-principles based calculations for confirmation of the transition states.

**Table 2:** Energy profile (kJ/mol) for the formation of cyclic oligomers through the AAM mechanism.

Entry	Reactions	ΔE_act_ *^a^*	ΔE *^b^*	ΔG_298K_ *^c^*	ΔG_450K_ *^c^*	ΔH_298K_ *^d^*	ΔH_450K_ *^d^*	If *^e^*
1	$T_{3}$ → $C_{3}$ + $H_{2}O$	131.9	−11.0	−17.1	−19.5	−12.6	−12.1	−365.4
2	$T_{4}$ → $C_{4}$ + $H_{2}O$	141.8	−8.0	−8.7	−11.1	−3.7	−4.2	−455.4
3	$T_{5}$ → $C_{5}$ + $H_{2}O$	140.3	−7.8	−26.3	−35.8	−8.2	−7.1	−381.8
4	$T_{6}$ → $C_{6}$ + $H_{2}O$	142.3	−4.3	−13.5	−18.6	−3.8	−3.1	−470.3
5	$T_{4}$ → $C_{3}^{1}$ + $H_{2}O$	130.6	−12.1	−13.8	−16.0	−9.5	−9.8	−316.9
6	$T_{5}$ → $C_{4}^{1}$ + $H_{2}O$	140.4	−11.8	−10.5	−11.1	−9.2	−9.8	−412.1
7	$T_{6}$ → $C_{5}^{1}$ + $H_{2}O$	144.9	−7.3	−3.4	−3.0	−4.0	−4.3	−321.6
8	$T_{7}$ → $C_{6}^{1}$ + $H_{2}O$	140.9	1.1	−24.3	−36.1	−2.0	−0.3	−389.9
9	$T_{5}$ → $C_{3}^{2}$ + $H_{2}O$	141.8	−19.2	−20.7	−21.6	−18.8	−19.2	−488.0
10	$T_{6}$ → $C_{4}^{2}$ + $H_{2}O$	144.0	−16.5	−0.8	4.4	−10.6	−11.7	−298.8
11	$T_{7}$ → $C_{5}^{2}$ + $H_{2}O$	134.4	−5.0	−15.2	−19.7	−6.8	−5.8	−401.9
12	$T_{8}$ → $C_{6}^{2}$ + $H_{2}O$	140.4	0.8	−0.9	−5.2	7.5	7.5	−478.8

*^a^* The energy barrier for the oligomerization. *^b^* The exothermicity for the oligomerization. *^c^* The free energy change of the oligomerization at 298 and 450 K. *^d^* The entropy change of the oligomerization at 298 and 450 K. *^e^* Imaginary frequencies obtained from first-principles based calculations for the confirmation of the transition states.

**Table 3:** Energy profile (kJ/mol) for the formation of linear oligomers through the MAM mechanism.

Entry	Reactions	ΔE_act_ *^a^*	ΔE *^b^*	ΔG_298K_ *^c^*	ΔG_450K_ *^c^*	ΔH_298K_ *^d^*	ΔH_450K_ *^d^*	If *^e^*
1	$T_{1}$ + $T_{1}$ → $T_{2}$ + $H_{2}O$	159.2	−2.4	−11.9	−16.7	−2.9	−2.1	−997.8
2	$T_{1}$ + $T_{2}$ → $T_{3}$ + $H_{2}O$	152.2	−4.0	−11.8	−16.1	−3.8	−3.2	−864.3
3	$T_{1}$ + $T_{3}$ → $T_{4}$ + $H_{2}O$	150.5	−4.6	−10.9	−14.2	−4.7	−4.1	−876.6
4	$T_{1}$ + $T_{3}$ → $T_{3}^{1}$ + $H_{2}O$	153.1	−2.5	0.0	0.0	0.7	0.6	−852.4
5	$T_{1}$ + $T_{4}$ → $T_{5}$ + $H_{2}O$	152.0	−4.0	−27.3	−37.3	−8.3	−6.8	−847.8
6	$T_{1}$ + $T_{5}$ → $T_{6}$ + $H_{2}O$	152.3	−4.3	−20.4	−27.6	−6.6	−5.7	−843.3
7	$T_{1}$ + $T_{6}$ → $T_{7}$ + $H_{2}O$	154.9	0.8	−6.2	−9.5	−0.3	0.5	−947.3
8	$T_{1}$ + $C_{3}$ → $C_{3}^{1}$ + $H_{2}O$	155.9	−4.8	−18.7	−25.2	−6.4	−5.4	−803.1
9	$T_{1}$ + $C_{4}$ → $C_{4}^{1}$ + $H_{2}O$	153.4	−3.4	−16.3	−21.9	−5.9	−4.8	−934.2
10	$T_{1}$ + $C_{5}$ → $C_{5}^{1}$ + $H_{2}O$	157.6	−1.5	−11.3	−16.8	−1.1	−0.3	−906.3
11	$T_{1}$ + $C_{6}$ → $C_{6}^{1}$ + $H_{2}O$	151.9	2.1	−11.2	−18.0	1.9	2.7	−859.2
12	$T_{1}$ + $C_{3}^{1}$ → $C_{3}^{2}$ + $H_{2}O$	155.0	−3.3	−8.4	−11.6	−2.2	−1.8	−802.5
13	$T_{1}$ + $C_{4}^{1}$ → $C_{4}^{2}$ + $H_{2}O$	154.0	0.6	−5.7	−9.3	1.1	1.6	−954.7
14	$T_{1}$ + $C_{5}^{1}$ → $C_{5}^{2}$ + $H_{2}O$	157.1	−14.5	−31.2	−37.0	−20.5	−19.0	−841.3
15	$T_{1}$ + $C_{6}^{1}$ → $C_{6}^{2}$ + $H_{2}O$	156.2	5.0	−10.8	−17.6	2.0	3.4	−760.5

*^a^* The energy barrier for the oligomerization. *^b^* The exothermicity for the oligomerization. *^c^* The free energy change of the oligomerization at 298 and 450 K. *^d^* The entropy change of the oligomerization at 298 and 450 K. *^e^* Imaginary frequencies obtained from first-principles based calculations for confirmation of the transition states.

**Table 4:** Energy profile (kJ/mol) for the formation of cyclic oligomers through the MAM mechanism.

Entry	Reactions	ΔE_act_ *^a^*	ΔE *^b^*	ΔG_298K_ *^c^*	ΔG_450K_^*c*^	ΔH_298K_ *^d^*	ΔH_450K_ *^d^*	If *^e^*
1	$T_{3}$ → $C_{3}$ + $H_{2}O$	136.5	−12.5	−14.6	−15.8	−12.3	−12.2	−793.3
2	$T_{4}$ → $C_{4}$ + $H_{2}O$	136.9	−16.9	−23.1	−26.7	−16.2	−16.0	−895.7
3	$T_{5}$ → $C_{5}$ + $H_{2}O$	150.9	−4.5	−17.0	−24.3	−2.0	−2.5	−861.9
4	$T_{6}$ → $C_{6}$ + $H_{2}O$	147.6	−3.6	−14.7	−20.5	−4.0	−2.9	−913.5
5	$T_{4}$ → $C_{3}^{1}$ + $H_{2}O$	133.5	−12.8	−9.8	−9.9	−9.3	−10.11	−928.3
6	$T_{5}$ → $C_{4}^{1}$ + $H_{2}O$	145.7	−8.8	−0.8	0.2	−2.4	−3.3	−828.0
7	$T_{6}$ → $C_{5}^{1}$ + $H_{2}O$	147.2	−7.3	−3.4	−3.0	−4.0	−4.3	−830.2
8	$T_{7}$ → $C_{6}^{1}$ + $H_{2}O$	145.8	−11.7	−18.6	−21.1	−14.3	−13.2	−807.2
9	$T_{5}$ → $C_{3}^{2}$ + $H_{2}O$	147.8	−18.8	−13.0	−10.6	−17.7	−17.8	−884.9
10	$T_{6}$ → $C_{4}^{2}$ + $H_{2}O$	146.0	−20.5	−24.7	−26.5	−21.4	−20.9	−739.1
11	$T_{7}$ → $C_{5}^{2}$ + $H_{2}O$	146.7	−19.4	−30.8	−34.6	−23.8	−22.7	−931.1
12	$T_{8}$ → $C_{6}^{2}$ + $H_{2}O$	146.5	−18.1	−20.0	−21.1	−18.5	−17.2	−837.3

*^a^* The energy barrier for the oligomerization. *^b^* The exothermicity for the oligomerization. *^c^* The free energy change of the oligomerization at 298 and 450 K. *^d^* The entropy change of the oligomerization at 298 and 450 K. *^e^* Imaginary frequencies obtained from first-principles based calculations for confirmation of the transition states.

## References

[1] Liang J., Liang Z.B., Zou R.Q., Zhao Y.L. (2017). Heterogeneous catalysis in zeolites, mesoporous silica, and metal-organic frameworks. Adv. Mater..

[2] Kulkarni A.R., Zhao Z.J., Siahrostami S., Norskov J.K., Studt F. (2018). Cation-exchanged zeolites for the selective oxidation of methane to methanol. Catal. Sci. Technol..

[3] Dusselier M., Davis M.E. (2018). Small-pore zeolites: Synthesis and catalysis. Chem. Rev..

[4] Ennaert T., Van Aelst J., Dijkmans J., De Clercq R., Schutyser W., Dusselier M., Verboekend D., Sels B.F. (2016). Potential and challenges of zeolite chemistry in the catalytic conversion of biomass. Chem. Soc. Rev..

[5] Mansir N., Taufiq-Yap Y.H., Rashid U., Lokman I.M. (2017). Investigation of heterogeneous solid acid catalyst performance on low grade feedstocks for biodiesel production: A review. Energy Conv. Manag..

[6] Goh P.S., Ismail A.F. (2018). A review on inorganic membranes for desalination and wastewater treatment. Desalination.

[7] Burakov A.E., Galunin E.V., Burakova I.V., Kucherova A.E., Agarwal S., Tkachev A.G., Gupta V.K. (2018). Adsorption of heavy metals on conventional and nanostructured materials for wastewater treatment purposes: A review. Ecotox. Environ. Safe..

[8] Bhatnagar A., Sillanpaa M. (2017). Removal of natural organic matter (nom) and its constituents from water by adsorption - a review. Chemosphere.

[9] Goh P.S., Ismail A.F., Sanip S.M., Ng B.C., Aziz M. (2011). Recent advances of inorganic fillers in mixed matrix membrane for gas separation. Sep. Purif. Technol..

[10] Vermeiren W., Gilson J.P. (2009). Impact of zeolites on the petroleum and petrochemical industry. Top. Catal..

[11] Smit B., Maesen T.L.M. (2008). Towards a molecular understanding of shape selectivity. Nature.

[12] Cejka J., Centi G., Perez-Pariente J., Roth W.J. (2012). Zeolite-based materials for novel catalytic applications: Opportunities, perspectives and open problems. Catal. Today.

[13] Kabalan I., Lebeau B., Nouali H., Toufaily J., Hamieh T., Koubaissy B., Bellat J.-P., Daou T.J. (2016). New generation of zeolite materials for environmental applications. J. Phys. Chem. C.

[14] Li S.Y., Li J.F., Dong M., Fan S.B., Zhao T.S., Wang J.G., Fan W.B. (2019). Strategies to control zeolite particle morphology. Chem. Soc. Rev..

[15] Verboekend D., Nuttens N., Locus R., Van Aelst J., Verolme P., Groen J.C., Perez-Ramirez J., Sels B.F. (2016). Synthesis, characterisation, and catalytic evaluation of hierarchical faujasite zeolites: Milestones, challenges, and future directions. Chem. Soc. Rev..

[16] Verboekend D., Perez-Ramirez J. (2011). Design of hierarchical zeolite catalysts by desilication. Catal. Sci. Technol..

[17] Fan F.T., Feng Z.C., Li C. (2010). Uv raman spectroscopic study on the synthesis mechanism and assembly of molecular sieves. Chem. Soc. Rev..

[18] Li J.Y., Corma A., Yu J.H. (2015). Synthesis of new zeolite structures. Chem. Soc. Rev..

[19] Li Y., Cao H., Yu J. (2018). Toward a new era of designed synthesis of nanoporous zeolitic materials. Acs Nano.

[20] Li Y., Yu J.H. (2014). New stories of zeolite structures: Their descriptions, determinations, predictions, and evaluations. Chem. Rev..

[21] Grand J., Awala H., Mintova S. (2016). Mechanism of zeolites crystal growth: New findings and open questions. Crystengcomm.

[22] Quesne M.G., Silveri F., de Leeuw N.H., Catlow C.R.A. (2019). Advances in sustainable catalysis: A computational perspective. Front. Chem..

[23] Coronas J. (2010). Present and future synthesis challenges for zeolites. Chem. Eng. J..

[24] Gascon J., Kapteijn F., Zornoza B., Sebastian V., Casado C., Coronas J. (2012). Practical approach to zeolitic membranes and coatings: State of the art, opportunities, barriers, and future perspectives. Chem. Mater..

[25] Van Speybroeck V., Hemelsoet K., Joos L., Waroquier M., Bell R.G., Catlow C.R.A. (2015). Advances in theory and their application within the field of zeolite chemistry. Chem. Soc. Rev..

[26] Catlow C.R.A., Coombes D.S., Pereira J.C.G. (1998). Computer modeling of nucleation, growth, and templating in hydrothermal synthesis. Chem. Mater..

[27] Pereira J.C.G., Catlow C.R.A., Price G.D. (1999). Ab initio studies of silica-based clusters. Part i. Energies and conformations of simple clusters. J. Phys. Chem. A.

[28] Pereira J.C.G., Catlow C.R.A., Price G.D. (1999). Ab initio studies of silica-based clusters. Part ii. Structures and energies of complex clusters. J. Phys. Chem. A.

[29] Pereira J.C.G., Catlow C.R.A., Price G.D. (1998). Silica condensation reaction: An ab initio study. Chem. Commun..

[30] Trinh T.T., Jansen A.P.J., van Santen R.A. (2006). Mechanism of oligomerization reactions of silica. J. Phys. Chem. B.

[31] Henschel H., Schneider A.M., Prosenc M.H. (2010). Initial steps of the sol-gel process: Modeling silicate condensation in basic medium. Chem. Mater..

[32] Hu H., Hou H., He Z., Wang B. (2013). Theoretical characterizations of the mechanism for the dimerization of monosilicic acid in basic solution. Phys. Chem. Chem. Phys..

[33] Schaffer C.L., Thomson K.T. (2008). Density functional theory investigation into structure and reactivity of prenucleation silica species. J. Phys. Chem. C.

[34] Tossell J.A. (2005). Theoretical study on the dimerization of si(oh)(4) in aqueous solution and its dependence on temperature and dielectric constant. Geochim. Cosmochim. Acta.

[35] Mora-Fonz M.J., Catlow C.R.A., Lewis D.W. (2005). Oligomerization and cyclization processes in the nucleation of microporous silicas. Angew. Chem.-Int. Edit..

[36] Mora-Fonz M.J., Catlow C.R.A., Lewis D.W. (2007). Modeling aqueous silica chemistry in alkali media. J. Phys. Chem. C.

[37] White C.E., Provis J.L., Kearley G.J., Riley D.P., van Deventer J.S.J. (2011). Density functional modelling of silicate and aluminosilicate dimerisation solution chemistry. Dalton Trans..

[38] Xiao Y.T., Lasaga A.C. (1996). Ab initio quantum mechanical studies of the kinetics and mechanisms of quartz dissolution: Oh- catalysis. Geochim. Cosmochim. Acta.

[39] Pelmenschikov A., Leszczynski J., Pettersson L.G.M. (2001). Mechanism of dissolution of neutral silica surfaces: Including effect of self-healing. J. Phys. Chem. A.

[40] Criscenti L.J., Kubicki J.D., Brantley S.L. (2006). Silicate glass and mineral dissolution: Calculated reaction paths and activation energies for hydrolysis of a q(3) si by h3o+ using ab initio methods. J. Phys. Chem. A.

[41] Zhang X.-Q., Trinh T.T., van Santen R.A., Jansen A.P.J. (2011). Mechanism of the initial stage of silicate oligomerization. J Am Chem Soc.

[42] Zhang X.-Q., van Santen R.A., Jansen A.P.J. (2012). Kinetic monte carlo modeling of silicate oligomerization and early gelation. Phys. Chem. Chem. Phys..

[43] McIntosh G.J. (2013). Theoretical investigations into the nucleation of silica growth in basic solution part ii - derivation and benchmarking of a first principles kinetic model of solution chemistry. Phys. Chem. Chem. Phys..

[44] McIntosh G.J. (2013). Theoretical investigations into the nucleation of silica growth in basic solution part i - ab initio studies of the formation of trimers and tetramers. Phys. Chem. Chem. Phys..

[45] Catlow C.R.A., Bromley S.T., Hamad S., Mora-Fonz M., Sokol A.A., Woodley S.M. (2010). Modelling nano-clusters and nucleation. Phys. Chem. Chem. Phys..

[46] Sefcik J., McCormick A.V. (1997). Thermochemistry of aqueous silicate solution precursors to ceramics. Aiche J..

[47] Mora-Fonz M.J., Catlow C.R.A., Lewis D.W. (2008). H-bond interactions between silicates and water during zeolite pre-nucleation. Phys. Chem. Chem. Phys..

[48] Putz M.V., Russo N., Sicilia E. (2004). On the applicability of the hsab principle through the use of improved computational schemes for chemical hardness evaluation. J. Comput. Chem..

[49] Putz M.V. (2008). Density functionals of chemical bonding. Int. J. Mol. Sci..

[50] Putz M.V. (2008). Maximum hardness index of quantum acid-base bonding. Match-Commun. Math. Comput. Chem..

[51] Putz M.V. (2011). Chemical action concept and principle. Match-Commun. Math. Comput. Chem..

[52] Becke A.D. (1988). A multicenter numerical-integration scheme for polyatomic-molecules. J. Chem. Phys..

[53] Lee C.T., Yang W.T., Parr R.G. (1988). Development of the colle-salvetti correlation-energy formula into a functional of the electron-density. Phys. Rev. B.

[54] Delley B. (1990). An all-electron numerical-method for solving the local density functional for polyatomic-molecules. J. Chem. Phys..

[55] Delley B. (2000). From molecules to solids with the dmol(3) approach. J. Chem. Phys..

[56] Klamt A., Schuurmann G. (1993). Cosmo–A new approach to dielectric screening in solvents with explicit expressions for the screening energy and its gradient. J. Chem. Soc. Perkin Trans..

[57] Baldridge K., Klamt A. (1997). First principles implementation of solvent effects without outlying charge error. J. Chem. Phys..

[58] Andzelm J., Kolmel C., Klamt A. (1995). Incorporation of solvent effects into density-functional calculations of molecular-energies and geometries. J. Chem. Phys..

[59] Govind N., Petersen M., Fitzgerald G., King-Smith D., Andzelm J. (2003). A generalized synchronous transit method for transition state location. Comput. Mater. Sci..

[60] Kelly C.P., Cramer C.J., Truhlar D.G. (2005). Sm6: A density functional theory continuum solvation model for calculating aqueous solvation free energies of neutrals, ions, and solute-water clusters. J. Chem. Theory Comput..

